# Effect of PCSK9 inhibitor on lipoprotein particles in patients with acute coronary syndromes

**DOI:** 10.1186/s12872-020-01827-0

**Published:** 2021-01-07

**Authors:** Tingting Li, Yingyi Zhang, Hongliang Cong

**Affiliations:** 1 Tianjin Medical University, Department of Cardiology, Tianjin Chest Hospital, Tianjin, China; 2 Department of Cardiology, Tianjin Chest Hospital, No. 261 Taierzhuang South Road, Jinnan District, Tianjin, China

**Keywords:** PCSK9 inhibitor, Nuclear magnetic resonance spectroscopy, Lipoprotein particles, Acute coronary syndromes, Low-density lipoprotein particle

## Abstract

**Background:**

To assess the effects of proprotein convertase subtilisin/kexin type 9 inhibitor (evolocumab) on lipoprotein particles subfractions with Nuclear Magnetic Resonance spectroscopy in patients with acute coronary syndromes.

**Methods:**

A total of 99 consecutive patients with ACS were enrolled and assigned to either the experimental group (n = 54) or the control group (n = 45). The combination therapy of PCSK9 inhibitor (Repatha®, 140 mg, q2w) and moderate statin (Rosuvastatin, 10 mg, qn) was administered in the experimental group, with statin monotherapy (Rosuvastatin, 10 mg, qn) in the control group. The therapeutic effects on lipoprotein particle subfractions were assessed with NMR spectroscopy after 8 weeks treatment, and the achievement of LDL-C therapeutic target in both groups were analyzed.

**Results:**

In the experimental group, after 8 weeks of evolocumab combination treatment, the concentrations of blood lipids (TC, LDL-C and its subfractions [LDL-1 to 6], VLDL-C and its subfractions [VLDL-1 to 5], IDL-C, and HDL-C), lipoprotein particles, and their subfractions [VLDL-P, IDL-P, LDL-P, and its subfractions [LDL-P1 to 6], apoB, and LP(a)] demonstrated therapeutic benefits with statistical significance (*P* < 0.05). The decrease in total LDL-P concentrations was mainly due to a decreased concentration of small-sized LDL particles (LDL-P 5 + 6), which was significantly more prominent than the decrease in medium-sized LDL-P (LDL-P3 + 4) and large-sized LDL-P (LDL-P1 + 2) (*P* < 0.001). According to lipid control target recommended by the latest China Cholesterol Education Program Expert Consensus in 2019, after 8 weeks treatment, 96.3% patients in the experimental group and 13.3% in the control group had achieved the LDL-C therapeutic target (*P* < 0.01).

**Conclusions:**

Evolocumab combination treatment for 8 weeks significantly improves the plasma lipid profiles in ACS patients, and significantly decrease the concentration of lipoprotein particles which might contribute to the pathonesis of atherosclerosis.

## Background

Low-density lipoprotein cholesterol (LDL-C) is the primary target of therapy in patients with acute coronary syndromes (ACS) in guidelines worldwide [1, 2]. Recent clinical trial evidence confirms that there is substantial incremental reduction in risk for acute cardiovascular events when LDL-C is reduced to very low levels (< 50 mg/dl) in patients with established coronary artery disease, however, risk for events among these treated patients remained substantial [3–6]. LDL is a heterogeneous lipoprotein fraction comprising different LDL subclasses that vary in density, size and composition due to continuous remodeling of lipoproteins in the blood [7], whose chemical components and physiologic functions differ a lot from each other. LDL particles of different sizes might not play the same role in the progression of atherosclerosis [8], indicating that the subfractions of LDL particles closely correlates with their functions, which is of grander clinical significance [9–11].

Statins are the first choice for lipid-lowering agents in clinical applications, which could also effectively decrease the risk of cardiovascular diseases. Nonetheless, studies revealed that even with high-dose statins, cardiovascular events were still of elevated incidence in high-risk patients. Furthermore, some patients show poor tolerance for high-dose statin therapy. In recent years, novel lipid-lowering agents, proprotein convertase subtilisin/kexin type 9(PCSK9) inhibitors, are receiving more attention, and impressive progresses were made in relevant studies. With the conclusion of a series of clinical trials, PCSK9 inhibitors are gradually proven to be effective in lowering blood lipid levels and preventing cardiovascular diseases [12].

The aim of this study was to evaluate the effect of statins, the traditional lipid-lowering agents, on lipoprotein particles subfractions. In addition, the effect of PCSK9 inhibitor (Repatha®), a novel lipid-lowering agent, on lipoprotein particles subfractions will also be explored. It has been demonstrated that statins in combination with PCSK9 inhibitor can further decrease LDL-C level by 50–70% [13], bringing greater cardiovascular benefit for patients. It remains unclear whether this benefit is attributed to an overall decrease in LDL-C or the decrease in certain lipoprotein subfractions, which would be preliminary discussed in this study.

## Methods

### Study population

The study was approved by the Medical Ethics Committee of Tianjin Chest Hospital. Informed consents were obtained from all subjects. ACS patients who presented to the Tianjin Chest Hospital from May to December 2019 were enrolled, with the inclusion criteria as follows: (1) Informed consent obtained from the participant who voluntarily take the medications, and related documents signed; (2) Patients aged 18 years and older presenting within 72 h after pain onset associated with either one of the following primary diagnoses: ST-elevation myocardial infarction (STEMI), non-ST-elevation myocardial infarction (NSTEMI) or unstable angina pectoris (UAP). The diagnosis of ACS patients follows the emergency rapid diagnosis and treatment guidelines for acute coronary syndrome (2019) [14]; (3) Patients who have a clearly documented LDL-C level higher than 2.6 mmol/l(100 mg/dl) before taking any medication or an LDL-C level higher than 1.8 mmol/l (70 mg/dl) while on lipid-lowering agents. The exclusion criteria include: (1) Severe end-stage diseases, such as renal dysfunction(eGFR < 30 ml/min/1.73 m^2^), heart failure (left ventricular ejection fraction < 35%), and malignant carcinoma, and liver dysfunction (liver enzymes ≥ 3times the upper limit of normal); (2) Systematic inflammatory disease or severe infection; (3) Thyroid disorder; (4) Pregnant or lactating women; (5) Suspected alcohol or other substance abuse, or other conditions that might impair follow-up or complicate subsequent treatment, deemed by the researcher, e.g. patients with frequent changes of workplace that might become lost on follow-up.

The 99 enrolled ACS patients were divided into two groups according to their medication: 54 in the experimental group receiving the combination therapy of PCSK9 inhibitor (Repatha®, 140 mg, q2w) and moderate statin (rosuvastatin, 10 mg, qn), and 45 in the control group receiving statin monotherapy (rosuvastatin, 10 mg, qn).

### Blood sample collection and lipid measurements

At baseline and after 8 weeks treatment, the participants in the two groups were collected peripheral venous blood at the fasting and resting state in the morning for examination.

Routine blood lipid profile measurements: The participants were fasted for 8 h. The blood was collected in a serum tube containing an inert separating gel. After the blood was fully coagulated, it was centrifuged at 3000 rpm for 10 min, and the supernatant was taken for testing. Lipid profile parameters (concentrations of TC, HDL-C, LDL-C, and TG) were measured using Roche c701 automated clinical chemistry analyzer. Apolipoprotein A1 (Apo-A1) and Apolipoprotein B (Apo-B) were measured by immunoturbidimetric methods. Plasma Lp(a) levels were determined by means of latex enhanced immuno-turbidimetry.

Nuclear magnetic resonance (NMR) spectroscopy measurements: 4 ml of the participant's whole blood (fasting for 8 h, BD blood vessels containing EDTA-K2 anticoagulant) was collected, centrifuged at 1700*g* for 13 min, and transferred the upper plasma into the cryopreserved tube, and then frozen and stored in aliquots at − 80 °C. During the test, the samples were taken out of the refrigerator, and after thawing completely, 400 μl plasma was taken and mixed with NMRS lipid buffer (Bruker Biospin, USA) 1:1, fully mixed, and then placed in a 5 mm NMR tube, and loaded into an automatic sample injector for testing [15].

Aliquots were measured blinded to patients' data by means of numbered codes and then merged with the clinical data set.

### Nuclear magnetic resonance (NMR) spectroscopy methods and testing program

According to the standard operating procedure of AVANCE IVDr magnetic resonance spectrometer system (Bruker Biospin) [16, 17]. The spectra were normalized to the same quantitative scale using Bruker’s QuantRef manager within TopSpin which is based on the PULCON method; hence, the spectral intensity is normalized to proton concentration in units of millimoles per liter. For data analysis, the study selected the commercial Bruker IVDr LIpoprotein Subclass Analysis (B.I.-LISA) method [18] as lipoprotein distribution prediction method, which used a PLS-2 regression model as the algorithm for spectral deconvolution [19]. This model provides information on main lipoprotein classes, including very low density lipoprotein (VLDL), intermediate density lipoprotein (IDL), LDL, and high density lipoprotein (HDL), as well as the five VLDL subfractions (VLDL-1 to VLDL-5), six LDL subfractions (LDL-1 to LDL-6), and four HDL subfractions (HDL-1 to HDL-4). Subfractions were sorted according to their increasing density and decreasing size in ascending order, respectively.

### Statistical analyses

Statistical analyses were performed by SPSS 22.0. Basic parameters of descriptive statistics for the analysed continuous variables are showed as a mean and standard deviation (SD) for normal distribution or as a median of the first and third quartiles (Q1–Q3) for a distribution other than normal. Comparisons of groups regarding continuous variables are tested with the *t* Student test (for a normal distribution of variables) or with the Mann–Whitney U test for nonnormal distributions. The categorical variables were presented as numbers (percentages), and comparisons between groups were conducted with Chi-square test. The concentrations of large-size LDL-P, medium-size LDL-P and small-size LDL-P were compared using ANOVA. Associations were examined by Pearson’s correlational analyses. The interval of two-sided *P* < 0.05 was considered statistically significant.

## Results

### Baseline data

The baseline data of the two groups was shown in Table 1.

**Table1:** Baseline clinical charateristics of the two groups and the comparisons of baseline blood lipid profiles and lipoprotein particles between them

Variable	PCSK9i + statins (n = 54)	Statins (n = 45)
Age (years), mean ± SD	60.6 ± 10.1	58.6 ± 10.6
Men, n (%)	34(63.0%)	30(66.7%)
Hypertension, n (%)	32(59.3%)	26(57.8%)
Diabetes, n (%)	15(27.8%)	10(22.2%)
Stroke history, n (%)	6(11.1%)	5(11.1%)
Dislipidemia, n (%)	25(46.3%)	20(44.4%)
MI history, n (%)	11(20.1%)	10(22.2%)
Prior PCI or CABG, n (%)	20(37.0%)	18(40.0%)
Family history of CAD, n (%)	17(31.5%)	13(28.9%)
Smoking, n (%)	22(41.1%)	23(51.1%)
Drinking, n (%)	20(37.0%)	16(35.6%)
BMI (kg/m^2^), mean ± SD	26 ± 5.0	27 ± 5.4
High waist circumference (women > 88 cm, men > 102 cm), n(%)	14(25.9%)	13(28.9%)
Statin use, n (%)	21(38.9%)	17(37.7%)
ACS type, n (%)		
STEMI	10(18.5%)	12(26.7%)
NSTEMI	24(44.4%)	20(44.4%)
UAP	20(37.0%)	13(28.9%)
Total cholesterol (mg/dl), mean ± SD	214.2 ± 42.4	189.7 ± 36.5
LDL-C (mg/dl), mean ± SD	123.7 ± 32.3	103.7 ± 28.1
IDL-C (mg/dl), mean ± SD	15.5 ± 9.1	13.6 ± 7.6
VLDL-C (mg/dl), mean ± SD	31.9 ± 17.9	25.3 ± 15.5
HDL-C (mg/dl), mean ± SD	43.9 ± 7.8	44.3 ± 9.4
Lp(a) (nmol/l), median (Q1, Q3)	73.1(13.7,102.3)	32.7(8.2,49.1)
ApoB (mg/dl), mean ± SD	108.7 ± 24.2	93.0 ± 20.8
LDL-P total (nmol/l), mean ± SD	1573.9 ± 375.3	1317.8 ± 337.8
VLDL-P (nmol/l), mean ± SD	237.5 ± 106.0	182.8 ± 89.2
IDL-P (nmol/l), mean ± SD	91.0 ± 47.7	88.6 ± 43.3

*PCI* percutaneous coronary intervention, *CABG* coronary artery bypass graft, *BMI* body mass index, *STEMI* stsegment elevation myocardial infarction, *NSTEMI* non-ST-segment elevation myocardial infarction, *UAP* unstable angina pectoris, *LDL-C* low-density lipoprotein cholesterol, *IDL-C* intermediate-density lipoprotein, *VLDL-C* very low-density lipoprotein cholesterol, *HDL-C* high-density lipoprotein cholesterol, *Lp(a)* Lipoprotein a, *ApoB* Apolipoprotein B, *LDL-P* low-density lipoprotein particle concentration, *VLDL-P* very low-density lipoprotein particle concentration, *IDL-P* intermediate-density lipoprotein particle concentration, *Q1, Q3* fifirst and third quartiles, *SD* standard deviation

Demographic data, clinical characteristics, baseline blood lipid profiles, and lipoprotein particle concentrations of the 99 participants were shown in Table 1. 63.0% and 66.7% were males in the experimental group and control group, respectively; while 27.8% and 22.2% were diabetic, respectively. No significant difference was found in age, gender, statin use, comorbidities including hypertension, stroke history, dislipidemia, diabetes mellitus, MI history, prior PCI or CABG, high waist circumference and body mass index (BMI) between the two groups (*P* > 0.05). The comparisons of the prevalence of STEMI, NSTEMI, and UAP revealed no significant difference (*P* > 0.05). At baseline, the median and interquartile range of LP(a) levels in the two groups were 73.1 (13.7, 102.3) and 32.7 (8.2, 49.1), respectively, significantly higher in the experimental group with a P value of 0.032. Other baseline lipid profiles and lipoprotein particle concentrations were comparable between the two groups. The LDL-C concentrations in the experimental and control groups were 123.7 ± 32.3 mg/dl and 103.7 ± 28.1 mg/dl, respectively; and LDL-P concentrations were 1573.9 ± 375.3 nmol/l and 1317.8 ± 337.8 nmol/l, respectively. Other baseline characteristics of blood lipid profiles and lipoprotein particles were shown in Table 1.

### Correlations between NMR spectroscopy and enzymatic method in the measurement of lipid parameters

Pearson correlation analyses were performed to assess the consistency between the NMR spectroscopy and enzymatic method measurements, in terms of the six items that they had in common (triglycerides [TG], total cholesterol [TC], LDL-C, high-density lipoprotein [HDL-C], Apo-A1, and Apo-B). The correlation coefficient was between 0.839 and 0.912, indicating close correlation between the two sets of measurement (Table 2).

**Table 2:** Pearson coefficient of correlation in lipid parameters measured by NMR spectroscopy and enzymatic method

	R values	*P* values
TG	0.849	0.000
TC	0.912	0.000
HDL-C	0.858	0.000
LDL-C	0.839	0.000
Apoa1	0.865	0.000
ApoB	0.854	0.000

### Changes in lipid profiles after treatment in the two groups

In the experimental group, after a combined treatment of evolocumab and moderate statins for 8 weeks, benefits in LDL-C concentrations and other blood lipids parameters were revealed with statistical significance (*P* < 0.05). TC, LDL-C and its subfractions (LDL-1 to -6), VLDL-C and its subfractions (VLDL-1 to -5), and IDL-C significantly decreased compared to baseline (*P* < 0.001). The decrease in total LDL-C concentrations was mainly due to a decreased concentration of small-sized LDL particles (LDL 5 + 6). In contrast, a significant increase in HDL-C concentrations was observed (*P* < 0.05), which could be mainly attributed to an increase in small-sized HDL particles (HDL 3 + 4). The concentrations of TC, LDL-C, VLDL-C and IDL-C were reduced by 48.4%, 65.5%, 26.3%, and 62.0%, respectively, while the HDL-C concentrations was increased by 7.6%, compared to baseline.

After 8 weeks of single moderate statins treatment in the control group, the concentrations of TC, IDL-C, and LDL-C significantly decreased by 16.9%, 20.7%, and 18.1%, respectively (*P* < 0.05). But the concentrations of VLDL-C and HDL-C did not decrease significantly after treatment (*P* > 0.05).

After 8 weeks treatment, the absolute reduction in the concentrations of TC, LDL-C, VLDL-C, and IDL-C in the experimental and control groups were (14.7 ± 15.4 vs. 5.2 ± 12.9), (81.6 ± 28.7 vs. 22.9 ± 25.1), (14.7 ± 15.4 vs. 5.2 ± 12.9), and (10.0 ± 7.9 vs. 4.3 ± 6.4), respectively, with significant differences between the two groups (*P* < 0.05).

### Changes in lipoprotein particle concentrations after treatment in the two groups

Changes in lipoprotein particle concentrations were presented in Table 3. After 8 weeks of combined treatment of evolocumab and moderate statins, statistically significant benefits in the concentrations of LDL-P and other lipoprotein particle concentrations were observed (*P* < 0.05). VLDL-P, IDL-P, LDL-P and its subfractions (LDL-P1 to 6), ApoB and LP(a) all decreased compared to baseline (*P* < 0.001). The concentration of total LDL-P before and after treatment was 1573.9 ± 375.3 and 463.6 ± 246.7 respectively, showing a reduction of 71.1% (*P* < 0.001). The decrease in total LDL-P concentrations was mainly due to a decreased concentration of small-sized LDL particles (LDL 5 + 6). In this study, LDL-P were further classified into large (LDL-P1 + 2), medium (LDL-P3 + 4) and small LDL-P (LDL-P5 + 6) by the size of the particles. The concentrations of small-sized LDL-P decreased by 76.8%, with a significantly larger extent than medium-sized and large-sized LDL-P (*P* < 0.001). The concentrations of VLDL-P, IDL-P, LP(a), and apoB decreased by 20%, 53.3%, 21%, and 60.9%, respectively, while the size of LDL-P in the experimental group increased by 3.6% (*P* < 0.001), as compared to an insignificant change in the control group.

**Table 3:** Absolute and relative reduction in lipid levels and lipoprotein particle concentration compared to baseline after 8 weeks treatment

	Evolocumab + statins			Statins		
	Week 8	Change from baseline	Percent change from baseline (%)	Week 8	Change from baseline	Percent change from baseline (%)
VLDL-C (mg/dl) Total	17.2 ± 7.3	14.7 ± 15.4	26	20.0 ± 12.1	5.2 ± 12.9	7.9
VLDL1	6.7 ± 4.6	6.1 ± 9.1	26.3	6.5 ± 4.5	3.5 ± 6.6	21.6
VLDL2 + 3	2.6 ± 1.5	2.8 ± 2.7	20.4	3.6 ± 2.5	0.6 ± 2.3	− 20
VLDL4 + 5	1.9 ± 1.3	2.2 ± 2.5	40.9	2.9 ± 2.3	0.6 ± 1.7	2.8
IDL-C (mg/dl)	5.7 ± 4.1	10.0 ± 7.9	62.0	9.3 ± 4.7	4.3 ± 6.4	20.7
LDL-C (mg/dl) Total	43.8 ± 21.8	81.6 ± 28.7	65.3	80.7 ± 14.8	22.9 ± 25.1	18.1
LDL1 + 2	10.4 ± 4.3	9.6 ± 8.5	40.4	11.3 ± 4.6	4.2 ± 5.7	21.2
LDL3 + 4	7.0 ± 4.9	10.2 ± 9.4	50.3	11.1 ± 4.5	2.4 ± 5.9	− 20.7
LDL5 + 6	5.0 ± 5.6	21.2 ± 12.7	79.5	17.6 ± 5.9	5.4 ± 7.4	6.6
HDL-C (mg/dl) Total	46.9 ± 8.3	− 2.9 ± 6.5	− 7.6	44.8 ± 8.3	− 0.4 ± 9.0	− 3.4
HDL1 + 2	10.2 ± 4.4	0.7 ± 3.6	− 2.2	10.5 ± 4.2	0.1 ± 2.6	− 2.7
HDL3 + 4	12.6 ± 5.4	− 1.9 ± 3.2	− 82.7	11.3 ± 5.3	− 0.6 ± 4.1	27.2
TC (mg/dl)	109.9 ± 27.9	106.0 ± 37.8	48.4	153.7 ± 18.4	35.9 ± 33.7	16.9
VLDL-P (nmol/l)	158.2 ± 56.9	80.2 ± 84.8	19.1	165.5 ± 80.3	17.4 ± 79.5	− 1.2
IDL-P (nmol/l)	37.4 ± 22.1	54.0 ± 45.2	53.3	60.7 ± 28.8	27.8 ± 31.9	24.5
LDL-P (nmol/l) Total	463.6 ± 246.7	1128.9 ± 374.6	71	985.2 ± 203.3	332.5 ± 32.6	21.7
LDL1 + 2	113.5 ± 40.1	91.4 ± 81.3	38.33	120.4 ± 45	40.5 ± 54.1	14.5
LDL3 + 4	80.1 ± 52.7	119.8 ± 98.9	57.3	129.5 ± 45	32.5 ± 67.3	− 16.7
LDL5 + 6	70.9 ± 76.8	304.8 ± 192.1	76.8	246.4 ± 91.8	83.0 ± 99.4	13.3
Lp(a) (nmol/l)	57.5(5.2,72.8)	15.6(8.5,29.5)	21.1	42.6(8.9,62.8)	− 13.9(− 19.2,− 0.3)	− 42.5
ApoB	42.3 ± 14.8	67.5 ± 23.6	60.9	71.5 ± 14.0	21.5 ± 20.2	20.8
LDL-C:HDL-C ratio	0.9 ± 0.4	2.0 ± 0.8	67.3	1.9 ± 0.5	0.5 ± 0.7	18.3
Apo-B:Apo-A1 ratio	0.3 ± 0.1	0.6 ± 0.2	63.3	0.6 ± 0.1	0.2 ± 0.2	21.4
LDL-P size	20.9 ± 0.5	− 0.7 ± 0.6	− 3.6	20.2 ± 0.2	0.0 ± 0.1	0.0

In the control group, after 8 weeks of statin monotherapy, IDL-P, LDL-P, and Apo-B concentrations significantly lowered compared to baseline (*P* < 0.05), but no significant change was found in VLDL-P concentration (*P* > 0.05). The concentrations of IDL-P, LDL-P and ApoB decreased by 24.5%, 21.7%, and 20.8% compared to baseline. Comparison of LDL-P subfractions (small-sized, medium-sized and large-sized) revealed no significant difference (*P* > 0.05). LP(a) was seen no significant decrease, instead an increase in the control group.

After 8 weeks treatment, the absolute reductions in the concentrations of VLDL-P, IDL-P, LDL-P and ApoB were significantly different between the experimental and control group (*P* < 0.05).

### The achievement of LDL-C therapeutic target in both groups

After 8 weeks treatment, the mean LDL-C concentration in the experimental group decreased from 124 to 44 mg/dl, showing a 65.3% reduction compared to the baseline, in contrast to a 18.1% reduction from 104 to 81 mg/dl in the control group. The difference in the percentage of reduction between the two groups was 47.2%, with an absolute difference of LDL-C concentration of 57 mg/dl.

**Fig. 1:**
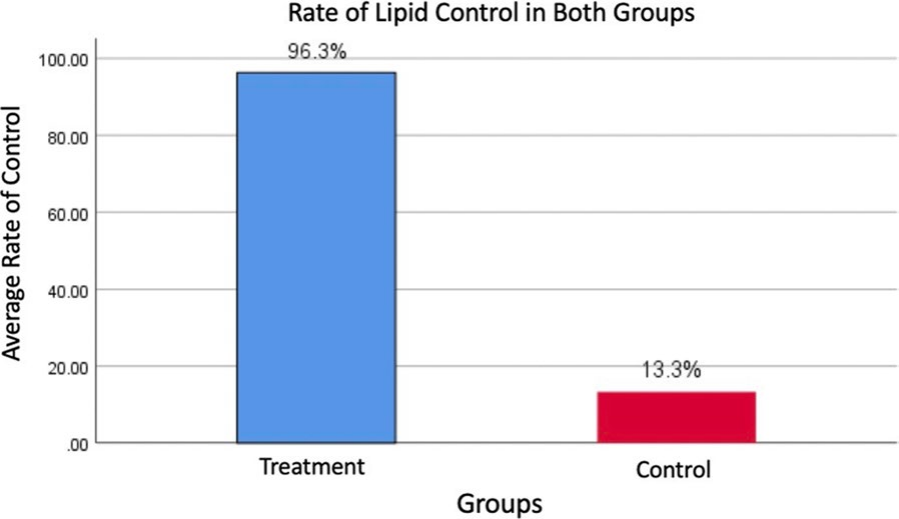
The achievement of LDL-C therapeutic target in both groups

According to the China Cholesterol Education Program (CCEP) Expert Consensus in 2019: for patients at very high risk, the target of LDL-C concentration should be lower than 55 mg/dl, or at least a 50% reduction from baseline. After 8 weeks treatment, 96.3% patients in the experimental group and 13.3% in the control group had achieved the LDL-C therapeutic target (*P* < 0.01) (Fig. 1).

## Discussion

PCSK9 inhibitor is a novel mechanism for reducing levels of LDL-C. Evolocumab, a fully human monoclonal antibody targeting human PCSK9, inhibits the binding of PCSK9 and LDL-R. Effective lipid modification could be achieved by PCSK9 inhibitors, manifesting as decreased serum TC, LDL-C, and non-high-density lipoprotein cholesterol, and increased HDL-C [20]. In addition, compared to statins, PCSK9 inhibitor exhibit stronger efficacy in lowering LDL-C and TC, as well as increasing HDL-C [21]. Compared to statin alone, a combination therapy of PCSK9 inhibitor and statin demonstrates more advantages in lowering LDL-C [22]. Although the effect of evolocumab on decreasing LDL-C is well characterized, little is known about its effects on lipoprotein particles subfractions in patients with ACS in Asia.

As we all know, lipoprotein particle play an important role in atherosclerosis, including LDL-P, as well as VLDL and IDL-P. The more LDL particles could increase the risk of atherosclerosis. It has been suggested that smaller, denser LDL particles are more atherogenic than larger particles [23]. Smaller denser LDL particles may be more atherogenic because they biophysically can more easily cross into the subendothelial space, their lipids are more prone to oxidation rendering them better substrates for macrophage scavenging, and they have lower affinity to the LDL receptor because of a change in the 3-dimensional conformation of apoprotein B [24, 25]. As remnant lipoproteins (small molecule VLDL and IDL) are also found in the atherosclerotic plaques, reduction of remnant lipoproteins is also important in addition to the reduction of LDL.

In the present study, we adopted NMR spectroscopy to assess the effects of evolocumab on blood lipid and lipoprotein particles subfractions in patients with acute coronary syndromes. Firstly, Table 1 show that baseline data were comparable between the two groups. Secondly, we analyze the correlations between NMR spectroscopy and enzymatic method in the measurement of lipid parameters. The correlation coefficient was between 0.839 and 0.912, indicating close correlation between the two sets of measurement. Then, we compared the effects of the two treatments on blood lipid and lipoprotein particles subfractions. Both LDL-C and LDL-P were reduced significantly compared with statin monotherapy. In our study, LDL-C concentration were decreased by 65.5% in combination treatment group, which is in agreement with ODYSSEY OUTCOMES trial [26]. The observed 71% reduction in total LDL-P concentration after 8 weeks of treatment with evolocumab combined moderate statins approximately matched the magnitude of previously reported reductions. In the Koren et al. [27] study, Alirocumab reduced mean concentrations of total LDL-P by 63.3% after 12 weeks treatment. A post hoc analysis from DESCARTES trial demonstrated that in patients receiving evolocumab, week 52 total LDL-P concentration decreased to 610 nmol/l, a treatment difference of 50% versus placebo [28].

In the study, LDL-P were further classified into large (LDL-P1 + 2), medium (LDL-P3 + 4) and small LDL-P (LDL-P5 + 6) by the size of the particles. The decrease in total LDL-P concentrations was mainly due to a decreased concentration of small-sized LDL particles (LDL 5 + 6). The percentage reduction in small-sized LDL-P was approximately twice that of large-size LDL-P (76.8% vs. 38.3%), which likely accounts for the increase in average LDL particle size. The results of our study was not consistent across literature [27]. The evolocumab combination treatment group substantially reduced triglyceride-enriched lipoprotein particles including VLDL-P 、 IDL-P by 20%and 53.3%, respectively. The HDL-C level was increased by 7.6%. ApoB, representing the level of circulating numbers of atherogenic lipoproteins, decreased by 60.9%. LP(a) decreased by 21%, which is in agreement with the previous studies.

It is well accepted that smaller size of lipoprotein particles leads to an increased risk for atherosclerosis [29, 30]. In this study, evolocumab exhibited significant advantages in lowering the concentrations of small lipoprotein particles. We found a markedly reduction magnitude in small-size LDL (LDL5 + 6) and VLDL (VLDL4 + 5), compared to medium-size and large-size particles. Meanwhile, in evolocumab combination treatment group, the LDL-P size before and after treatment were 20.2 ± 0.4 and 20.9 ± 0.5, respectively, suggesting an increase of 3.6% in the size of lipoproteins after treatment.

2019 CCEP Expert Consensus recommended that the target of lipid control for recent ACS patients, a super high risk population of ASCVD, should achievement: an LDL-C level of less than 55 mg/dl, or a reduction of LDL-C of at least 50% compared to baseline [31]. In this study, the treat-to-target rate of blood lipids with statin alone was as low as 13.3%. ACS patients have a very high risk of early cardiovascular, with over 30% of cardiovascular events and mortality occurring in the first 4 days, and over 50% occurring in the first 15 days [32]. Previous studies showed that the major cause of early ACS risks was the rupture of atherosclerotic plaques [33]. Therefore, the *2019 ESC Guidelines for the Management of Dyslipidemias* recommended that: for patients who present with an ACS and whose LDL-C levels are not at goal, despite already taking a maximally tolerated statin dose and ezetimibe, the addition of a PCSK9 inhibitor early after the event (during hospitalization for the ACS event if possible) should be considered, in order to obtain earlier cardiovascular benefits [34].

## Study limitations

There were some limitations in this study. First, the sample size was relatively small, and the duration was relatively short, making it difficult to evaluate the definitive clinical outcomes. Thus, future studies with larger sample size and longer follow-up period are needed to evaluate the effects of medications on clinical events and outcomes. Second, patients were not randomized. Evolocumab is still a self-funded drug in our country, the cost is more expensive. We will consider the financial situation of the patients. This could induce some bias in the selection of the sample. In conclusion, 8 weeks of PCSK9 inhibitor could significantly improve the plasma lipid profiles in ACS patients, and significantly decrease the atherogenic lipoproteins particles including LDL-P and remnant lipoproteins particles. Given the low treat-to-target rate of blood lipids in ACS patients on statin monotherapy, an additional PCSK9 inhibitor is recommended to be initiated as early as possible to obtain earlier cardiovascular benefits.

## Conclusion

In summary, evolocumab combination treatment for 8 weeks significantly improves the plasma lipid profiles in ACS patients, and significantly decrease the concentration of lipoprotein particles which might contribute to the pathonesis of atherosclerosis.

## Data Availability

The datasets used and/or analysed during the current study are available from the corresponding author on reasonable request.

## Abbreviations

NMR: Nuclear magnetic resonance
ACS: Acute coronary syndromes
PCSK9: Proprotein convertase subtilisin/kexin type 9
ASCVD: Atherosclerotic cardiovascular disease
PCI: Percutaneous coronary intervention
CABG: Coronary artery bypass graft
BMI: Body mass index
STEMI: ST-segment elevation myocardial infarction
NSTEMI: Non-ST-segment elevation myocardial infarction
UAP: Unstable angina pectoris
LDL-C: Low-density lipoprotein cholesterol
IDL-C: Intermediate-density lipoprotein cholesterol
VLDL-C: Very low-density lipoprotein cholesterol
HDL-C: High-density lipoprotein cholesterol
Lp(a): Lipoprotein a
ApoB: Apolipoprotein B
LDL-P: Low-density lipoprotein particle concentration
VLDL-P: Very low-density lipoprotein particle concentration
IDL-P: Intermediate-density lipoprotein particle concentration
Q1, Q3: Fifirst and third quartiles
SD: Standard deviation
